# Supplementary material for: Uniportal Video-Assisted Thoracoscopic Segmentectomy for Early-Stage Non-Small Cell Lung Cancer: Overview, Indications, and Techniques
Source: Cancers (Basel). 2024 Jun 26;16(13):2343. doi: 10.3390/cancers16132343 (PMC11240445; doi:10.3390/cancers16132343)
Supplement: Supplementary file 1 [file cancers-16-02343-s001.zip › cancers-3037405 supplementary materials.pdf]

cancers

MDPI

Supplementary Materials of: Uniportal Video-Assisted Thoracoscopic Segmentectomy for Early-Stage Non-Small Cell Lung Cancer: Overview, Indications and Techniques

Supplementary Video S1:
https://drive.google.com/file/d/1M1P6zgzMXm7OkH_Ojkc9kIYUbFWuuU1p/view
(accessed on 15 May 2024)

Supplementary Video S2:
https://drive.google.com/file/d/1A79nCKc2k5lMeWsGgG5En6s9gv4bRh6J/view
(accessed on 15 May 2024)

Supplementary Video S3:
https://drive.google.com/file/d/1RLfysTxwvJrgiFRAOCDXBOCwsRMbHsYA/view
(accessed on 15 May 2024)

Supplementary Video S4:
https://drive.google.com/file/d/1i-xlW21u3amNdjR4EA5JNtAGoNdVL_Yt/view
(accessed on 15 May 2024)

Supplementary Video S5:
https://drive.google.com/file/d/1I09UUyk-m6FJTXFJ1ERNtXI-sd8VD9lv/view
(accessed on 15 May 2024)
